# Bank Performance and Noninterest Income: Evidence from Countries in the Asian Region

**DOI:** 10.1007/s10690-021-09357-1

**Published:** 2022-01-12

**Authors:** Sherika Antao, Ajit Karnik

**Affiliations:** 1 Alumna, Middlesex University Dubai, Knowledge Park, Dubai, UAE; 2 Middlesex University Dubai, Knowledge Park, Dubai, UAE

**Keywords:** Noninterest income, Bank performance, Asia–Pacific region, GMM estimation, Determinants of bank risk, ZSCORE

## Abstract

Noninterest income (NII) is income generated by banks from sources other than interest payments. Studies conducted on the relationship between NII and bank risk for the USA and Europe have found that emphasis on income diversification lowers risk in European banks but exacerbates it in American banks. Current research on Asian banks has not led to a coherent view of the relationship between NII and bank risk. We employ data over 25 years for 24 Asian countries to examine this relationship. Using the GMM estimation approach we estimate equations for two time-periods, 1996–2007 and 2008–2018, to examine the NII-bank risk relationship in the presence of some controlling financial, macroeconomic and policy variables. Our results show that non-interest income worsens bank risk for all 24 countries as well as for sub-groups of countries. We also find that, by and large, economic growth improves bank risk while inflation above a threshold worsens it. Finally, our proxy measure for monetary policy improves bank risk though fiscal policy seems to have no effect.

## Introduction

Noninterest income is understood as income generated by banks from sources other than interest payments, the most common examples of which are service fees such as ATM fees and loan origination fees (Haubrich & Young, 2019). There has been a substantial expansion of off-balance sheet activities in many banks globally, indicating that banks have been diversifying, thus making non-interest income an important source of bank revenue. As interest margins have been observed to be narrowing in banks of numerous countries, there has been a tendency towards diversifying into non-interest sources of income (Heffernan, 2005).

Numerous studies have been conducted on the relationship between non-interest income, and bank profitability and/or risk. However, much of this literature is focused on Europe and the USA with relatively little research on the Asian sector (Lee et al., 2014). Hsieh et al. (2013) also state that studies in Asian banks are sparse and claim that theirs is one of the first studies using Asian data. European studies by Smith et al. (2003), Baele et al. (2007) and Lepitit et al. (2008) have generally shown a positive effect of non-interest income on profitability though there is a possibility that greater diversity in bank income may reduce it. Kohler (2014) in a detailed study of German banks finds evidence that the impact of non-interest income on bank stability depends on banks' overall business models. Specifically, it was shown that banks that focus on traditional services become more stable with an increasing share of non-interest income. Hahm (2008) finds for OECD countries that non-interest income does not have a significant effect on risk-adjusted returns. Studies on the US banking system (DeYoung and Rice (2004), DeYoung and Roland (2001), Stiroh (2004, 2006), Stiroh and Rumble (2006)) have found that an increase in non-interest income led to a greater profit variability, which worsened the banks’ risk-return tradeoff. It was also noted that bank risk was found to be strongly negatively correlated to non-interest income sources, specifically trading income.

The view put forward in this paper is that experiences of the USA and Europe may not necessarily hold for Asian countries. The main reason for this is that the characteristics of the banking system in Asia and the role played by it in their respective economies may be quite different than what we observe in the USA or Europe. We discuss these details below.

### Special Characteristics of Asian Banks

As emerging economies of the world have started to play an important role in the world economy and, given that many of such economies are in Asia, a study of Asian banks assumes importance. Behaviour of banks in Asia is likely to be different from that observed in the USA and Europe for three important reasons:

One, banks in Asia play an extremely important role in financing business activities of the private sector as has been documented in numerous studies (Lee et al., 2014; Salike & Ao, 2018). This is unlike in the USA where markets and non-bank financial institutions dominate (Elliott, 2014). Even though there is some similarity between banks in Europe and Asia, the role that governments play in the banking system is different with government-owned banks being common in Asia as well as the existence of more intrusive government directives in the functioning of banks (Elliott, 2014; Walsh, 2014).

Two, most banks in East Asia were badly affected by the 1997–1998 Asian financial crisis (Jeon and Miller, 2004) in the aftermath of which numerous regulatory changes were introduced in many countries such as Korea, Indonesia, Malaysia, Philippines and Thailand (Asian Development Bank, 2017). Banks in Indonesia have experienced significant structural changes after the crisis of 1997 in the form of deregulation which has increased competition especially for the largest bank in the country (Ashyari & Rokhim, 2020). In the aftermath of that crisis, Korean bank regulators have enacted laws to restructure and expand the operating income revenue to increase its diversification from conventional banking activities to non-bank activities (Baek et al., 2018). In Malaysia, banks have faced a proliferation of non-performing loans. To overcome the problems faced by the banks and, in light of their fragile health, the Malaysian central bank undertook a number of measures the most important of which was the merger of banks (Sufian, 2006).

And, three, changes have been continually taking place in the Asian banking sector the most notable of which has been liberalisation. Banks are being coaxed to develop new financial products to meet the demands of the market in order to become more competitive and expand the scale of business (Hsieh et al., 2013). Liberalisation of the banking sector and increasing competition have been observed in a few Asian countries which also makes a study of Asian banks important. Chinese banks are facing challenges of interest-rate liberalization as well as expansion of non-bank institutions which has encouraged them to employ innovative measures in the face of declining traditional sources of income (Bian et al., 2015). Banks in Taiwan have faced the pressure of competition as the government has allowed the number of banks to increase from 24 in 1991 to 41 in 2007 (Chen & Lin, 2010). Banks in Vietnam have also faced competition pressures after financial integration and restructuring of the banking system (Dang, 2020). Further, after a period of ineffective credit growth, the government directed banks to boost their non-lending segments which diverge from what Chow and Surti (2011) call “narrow utility banks” which are deemed to be non-risky. All of these developments have forced banks to look for alternative sources of income.

Banks in India and Japan represent two more instances of peculiarities not found in Europe or the USA. The Indian banking sector, which, for decades, was dominated by the presence of government owned banks, has seen changes and the sector is now composed of government owned banks, private Indian owned banks and foreign owned banks (Trivedi, 2015). Performance-wise as well there is a tremendous amount of unevenness in the banking sector with government owned banks accounting for the largest share of non-performing assets (Bawa et al., 2019). Despite the presence of the government in the banking sector, reforms in the sector have led to changes in market structure, and have introduced competition (Ahamed, 2017). This increasing competitiveness has led banks to explore nontraditional, noninterest banking activities. Japanese banks have been operating in an excessively low interest regime since the 1990s as a consequence of which interest incomes have fallen (Weistroffer, 2013). With economic growth and, hence, credit growth stagnating over a long period of time, banks have tried to compensate for the slowing down of interest earnings by trying to exploit noninterest sources of income, albeit with only limited success.

### Non-interest Income and Bank Performance in Asian Banks

As far as the role of non-interest income in the determination of bank performance in Asia is concerned, Lee et al. (2014) find that non-interest activities decrease profitability as well as increase risk for savings banks but may do the reverse for other kinds of banks such as investments banks; Salike and Ao (2018), report that profitability of banks is determined by factors internal and external to the bank and further that income diversification (which depends on non-interest income) tends to improve profitability; Hsieh et al. (2013) show that income diversification by itself does not improve bank stability but, when the model is extended to incorporate the effects of globalization and laws and regulations, income diversification improves bank stability.

Williams (2016) points out that Australian banks did not experience the same level of trauma as did the US banks in the context of the global financial crisis (GFC). The study found that, as in the case of many studies dealing with Europe and the USA, non-interest income is riskier than interest income. Bian et al. (2015) report that non-interest income has a negative but non-significant effect on risk efficiency of Chinese commercial banks but they also find that commission and fee income significantly reduces risk efficiency. The results of Chen and Lin (2010) show that diversification can improve profitability and reduce risk. Dang (2020) finds that fee income has a beneficial effect on profits and risk-adjusted profits. Baek et al. (2018) have reported results relevant to this paper. Their cross-section results show positive and strong indirect effect of non-interest income share on z-score (measure of bank risk) as well as on profitability. For their panel data model, they find similar results for fee incomes. Ahamed (2017) hypothesizes that a shift toward non-interest income activities increases profits and risk-adjusted profits of Indian banks and finds strong evidence to support the hypothesis.

### Contribution of this Study

In view of the special characteristics of Asian banks noted in subsection 1.1 above, a study of banks in Asia assumes importance. This is not to say that Asian banks have been neglected, but what makes our study different is that we lay particular emphasis on the possible effects of the global financial crisis (GFC) of 2008. There have, indeed, been studies focusing on banks in a single country (see, for example, Ahamed, 2017 on India; Baek et al., 2018 on South Korea; Bian et al., 2015 on China; Chen & Lin, 2010 on Taiwan) but relatively few looking at a sample of countries. Among the latter, only one has been carried out in the recent past (Salike & Ao, 2018) while other two are Hsieh et al. (2013) and Lee et al. (2014). The latter two studies consider data only till 2009 when many countries in Asia were still suffering from GFC and hence they are unable to examine the performance of banks in the post-GFC time period. While Salike and Ao (2018) do consider data available till 2015 they do not carry out a comparison of the pre- and post-GFC time periods. The possible reason could well be the inadequacy of data in the post-GFC period. The present study is being undertaken when enough years of data are available in the post-GFC period. Specifically, our models will seek to examine how bank performance has changed in the time period after the GFC. The key indicator of bank performance that we use is bank risk (as discussed by Kohler, 2014) and employed by a few Asian studies (Baek et al., 2018; Hsieh et al., 2013). We examine the role of non-interest income (along with other determinants) in explaining bank risk (as measured by z-score) over the time period 1996–2020 for a group of 24 countries. The novel contribution of our study is that we examine this for the years before the GFC, from 1996 to 2007 and then for the time period from 2008 to 2020.

A further novel aspect of our study is that it is being undertaken as the world and Asia is recovering from the COVID-19 pandemic. Even though the cases of COVID started to be reported in China from November 2019, these started to spread to the rest of the world only from 2020. Hence, as far as our data is concerned, it will show the effects of the pandemic only in the year 2020. While we will make comments about pandemic in relation to banks in Asia, it may not be possible to carry out a rigorous statistical exercise given that only one year of such data would be available.

Our dataset includes over 1000 banks, spread over 24 Asian countries. Not only is our entire dataset in panel form but the data for each country and for various groups of countries is in panel form as well. We exploit this aspect of our data by examining the role of non-interest income for all 24 countries as a whole and also for sub-groups of these countries. Our overall results point to a negative influence of non-interest income on bank risk which is at variance with at least some of the studies that have been carried out for Asian banks. This results holds for the entire set of countries as well as for sub-groups of countries.

The plan of the paper is as follows: in Sect. 2, we discuss our data, the theory related to the models we use and the methodology used for estimating our bank risk  equations. Section 3 presents the details of our empirical exercises, Sect. 4 presents a comparison of the results across the sub-groups of countries and discusses these, and Sect. 5 concludes the paper.

## Data and Methodology

Research in this area has sought to explain profitability of banks or bank risk by relating it to a variety of factors, some of which are financial factors related to the specific bank and others are macroeconomic and policy variables which affect all banks within a country. The equation that we specify is stated as follows:

i $${\text{Bank}}\;{\text{risk}} = {{f}}\left( {{\text{financial}}\;{\text{variables}},{\text{macroeconomic}}\;{\text{variables}},\;{\text{policy}}\;{\text{variables}}} \right)$$

We define bank risk in terms of the ZSCORE which evaluates the risk exposure of banks (Baek et al., 2018; Stiroh & Rumble, 2006) and is indicative of a bank’s “distance-to-default” (Chiaramonte et al., 2016). A higher value for ZSCORE indicates lower risk and higher stability as the bank has higher levels of profitability and equity with less variability in profitability (Engle et al., 2014). Following Kohler (2014), we define:

$$ZSCORE_{it} = \frac{{ROA_{it} + {\raise0.7ex\hbox{${TE_{it} }$} \!\mathord{\left/ {\vphantom {{TE_{it} } {TA_{it} }}}\right.\kern-\nulldelimiterspace} \!\lower0.7ex\hbox{${TA_{it} }$}}}}{{SDROA_{i} }}$$

where ROA_it_ = Return on Asset for bank i in time period t. TE_it_ = Total equity of bank i in time period t. TA_it_ = Total assets of bank i in time period t. SDROA_i_ = standard deviation of ROA of bank i, computed over the sample period.

It may be pointed out that ZSCORE can be computed in different ways depending on how the three components (ROA, TE/TA and SDROA) in its definition are measured. For instance, Houston et al. (2010) measure all three components as averages over their period of analysis while Berger et al. (2014) compute rolling averages averages of ROA and (TE/TA) as well as rolling standard deviation of ROA (SDROA) over previous 12 quarters. It should also be noted that other measures of bank stability/risk have been used in the literature, such as, bank capitalization, non-performing loans and risk-weighted capital-asset ratio among others (Jayakumar et al., 2019; Pradhan et al., 2019; Baek et al., 2018; Skala & Weill, 2018). While there can, indeed, be a debate regarding which measure of bank risk or bank stability is to be used, we make use of the ZSCORE in our exercises. Several advantages of using the ZSCORE have been listed. Boyd and Graham (1986) and Strobel (2011) point out that ZSCORE does not require strong assumptions about the distribution of assets. Chiaramonte et al. (2016) also note the simplicity in the computation of the measure as an important advantage but, more importantly, report on the ability of ZSCORE to forecast bank failures with an accuracy of 76% as also the ability to predict bank defaults three years in advance. The ZSCORE has become a very commonly used measure of bank risks. Morgan and Pontines (2014) have used it in their study of 193 countries listed in the Global Financial Development Database of the World Bank (World Bank, 2020) which covers countries in all continents. Finally, many studies (Chen & Lin, 2010; Bian et al., 2015; Baek et al., 2018; Ahamed, 2017; Pham et al., 2021) related to banks in Asia make use of the ZSCORE to measure bank risk when studying the effects of income diversification, which makes it possible to compare our results with these studies. Clearly, the ZSCORE has been used as a measure of bank risk/stability in diverse countries with widely differing institutional settings.

Table 1 lists out all the independent variables used in our exercises. The first group defines the financial variables, the second defines macroeconomic variables and the third defines policy variables.

**Table 1:** Variable descriptions

Variable abbreviation	Description	Unit of Measurement
*Dependent variable*		
ZSCORE	A measure of bank risk (Logged)	Units
*Independent variables: financial variables*		
NII	The ratio of non-interest income to operating income	Percent
TA	Total assets of each bank (Logged)	Million US Dollars
TAGR	Growth rate of total assets	Percent
LLPRATIO	The ratio of loan loss provisions to total assets	Percent
LOANRATIO	The ratio of loans of a bank in a current year to the total assets	Percent
EQUITYRATIO	The ratio of equity to total assets	Percent
DEPOSITRATIO	The ratio of bank deposits to total assets	Percent
NPL_TL	The ratio of non-performingg loans to total loans	Percent
*Independent variables: macroeconomic variables*		
GROWTH	Rate of growth of GDP	Percent
INFLATION	Difference betwwen the rate of inflation in a year and the average rate of inflation for the relevant time period	Units
*Independent variables: policy variables*		
MONEY	Ratio of broad money to GDP	Percent
GOVT_EXP	Ratio of government expenditures to GDP	Percent

Our variable of interest in the determination of ZSCORE is NII. Engle et al. (2014) note that nontraditional business activities could permit banks to circumvent capital regulations and allow increased risk-taking that traditional activities do not permit. This is likely to create agency problems that have been studied extensively in the literature. The reason why risk-taking increases is that non-interest income is often more volatile than interest income but not necessarily more profitable. (Stiroh, 2004; Stiroh & Rumble, 2006). If this is true, the coefficient of NII will likely be negative and make the banks more risky in the presence of rising NII.

Total assets (TA) is included as an independent variable to proxy bank size (Nguyen, 2012). Bank size allows availing of advantages of economies of scale and earn higher incomes and also provide protection from negative shocks during recessions (Salike & Ao, 2018) The annual growth rate of assets (TAGR) is expected to have a positive impact on the risks faced by banks (Ahamed, 2017). In order to control for the asset structures in each bank, the ratio of loans to total assets (LOANRATIO) is included since it detects differences in the asset portfolios of banks (Sanya & Wolfe, 2011; Stiroh & Rumble, 2006). Equity capital to total assets ratio (EQUITYRATIO) is expected to affect risk by allowing banks with higher capital amounts to absorb any negative shocks. Loan loss provisions to total assets ratio (LLPRATIO) is a measure for the quality of loans in a bank. Banks could use loan loss provisions as a means to smooth their revenues (Ahamed, 2017). The ratio of deposits to total assets (DEPOSITRATIO) is also included as control variable following Lee et al. (2014). Finally, ratio of non-performing loans to total loans (NPL_TL) represents asset quality following Salike and Ao (2018).

In addition to bank specific financial variables, we also include a few macroeconomic variables in our equations. As noted by Salike and Ao (2018), macroeconomic variables are factors that are not within the control of the banks’ management. We include two of the macroeconomic factors mentioned by Engle et al. (2014) and by Salike and Ao (2018), namely, rate of growth of GDP (GROWTH) and rate of inflation (INFLATION). High GDP growth tends to increase the demand for bank loans and will likely improve bank profitability and reduce bank risks. However, the effect of inflation is ambiguous (Salike & Ao, 2018). This is further elaborated by Dhal et al. (2011) who term the relationship between financial stability and inflation to be a very “contentious issue” and point out that financial stability may be affected only when inflation rises above a threshold level. In our analysis, we take the average rate of inflation during a given time period as the threshold level and compute the gap between actual inflation for each year and the average inflation for the relevant time period. Since we will be estimating separate regression equations for two time periods, one for before the GFC and one after the GFC, we compute the average rate of inflation for each country separately for each time period and then compute the gap for each year.

We also include two more macroeconomics variables which we call policy variables. Monetary policy is operated by the central bank of a country and a variety of instruments are used to operationalise it dependening on the objectives (Filardo & Genberg, 2010; Morgan, 2013). Given that the monetary policy instruments differ across countries, it is difficult to capture measures of the policy in one representative variable. However,it is possible to consider a proxy measure. As stated by Mathai (2020), monetary policy is understood as the central bank seeking to change the size of the money supply. Albertazzi et al. (2021) further note that the traditional transmission channel of monetary policy depends on the central bank’s control over the level of deposits via reserve requirements and the money multiplier. Hence, we take the ratio of broad money to GDP (MONEY) as a proxy measure of the monetary policy variable. Fiscal policy is the other means by which governments seek to manage the economy and involves the use of government spending and taxation (Horton and ElGanainy, 2020). We include in our analysis the ratio of government expenditure to income (GOVT_EXP) as a fiscal policy variable.

Our data on financial variables have been extracted from CapitalIQ (2020), a comprehensive database for banks while data on macroeconomic variables have been sourced from World Development Indicators of the World Bank (undated). The distribution of banks and the number of observations across the 24 countries is given in Table 2. It will be be noticed that we have defined the first group of countries as the broad Asia–Pacific region. The Asian Development Bank (1965) include Australia and New Zealand as regional members in its Charter. However, when we estimate equations for this group we do so with and without Australia and New Zealand. As in Salike and Ao (2018), the banks used in the study were a mix of commercial banks, savings banks, Islamic banks, housing and mortgage banks, rural banks and holding finance companies.

**Table 2:** Distribution of banks across 24 Asian countries

Country	Number of Banks	Number of Observations
*Asia/Pacific Developed Countries*		
1. Australia	59	1475
2. Hong Kong	42	1050
3. Japan	130	3250
4. New Zealand	25	625
5. Singapore	5	125
6. South Korea	107	2675
*East and South-east Asian Countries*		
7. Brunei	1	25
8. Cambodia	2	50
9. China	360	9000
10. Indonesia	65	1625
11. Lao PDR	1	25
12. Macau	2	50
13. Malaysia	38	950
14. Mongolia	2	50
15. Philippines	18	450
16. Taiwan	49	1225
17. Thailand	27	675
18. Vietnam	20	500
*South Asian Countries*		
19. Bangladesh	34	850
20. India	85	2125
21. Maldives	1	25
22. Nepal	10	250
23. Pakistan	24	600
24. Sri Lanka	15	375
Total	1122	28,050

The actual number of observations available for estimating the equations will be lower due to missing values

It can be seen from Table 2 that, for each country, we have a panel dataset and the entire dataset is made up of a panel of panels of 1122 banks with data spread over twenty-five years from 1996 to 2020. It must be mentioned that we do face the problem of missing data for some of the variables that we employ in our models. Hence, we are forced to work with an unbalanced panel and the total number of effective observations is much less than 28,050. It may be noted that we have divided the countries in our dataset into three sub-groups based on the categorization followed by Capital IQ.

### Estimation Strategy

The estimation strategy is to specify an equation that relates bank risk/stability as measured by ZSCORE to the set of explanatory or independent variables that have been listed in Table 1. The estimation technique we use is called the Generalised Method of Moments which we discuss in Appendix 1. However, the results presented below can be appreciated without reference to the technical aspects of the estimation procedure. The output given in Tables 4, 6, 8, 10 and 12 (in Appendix 2) can be followed with just some rudimentary knowledge of regression equations. The first 14 rows of each of the tables give the estimated coefficients (along with standard errors and significance levels), the 15th and 16th rows report the number of observations and the number of groups (i.e. banks) included in each equation, the 17th and 18th rows give information on the maximum number of time periods for which data are available for some banks, and the average number of time periods of data per bank. Number of instruments (row 19) is an aspect of the estimation procedure which tackles the problem of endogeneity (as a result of which the estimated coefficients are rendered unreliable), the F statistic (row 20) indiates how well the regression equation is performing and the last three rows evaluate the quality of the estimated equations.

## Empirical Exercises

Our empirical strategy involves estimating dynamic panel data models employing the two-step system GMM estimator. We estimate two equations-one for 1996–2007 and the other for 2008–2020-for (a) all countries together (b) group of Asia/Pacific developed countries excluding Australia and New Zealand (c) group of Asia/Pacific developed countries including Australia and New Zealand (d) group of East and South-east Asian countries and (e) South Asian countries.

### All Countries

In this sub-section we look at the performance of all banks in our dataset spread across all 24 countries. We first provide, in Table 3, a brief description of our data, separately for the two time periods that we focus on.

**Table 3:** Descriptive Statistics (a) 1996–2007 (b) 2008–2020

	Frequencies	Mean	S.D	Min	Max
1996–2007					
ZSCORE	3326	2.24	1.82	0.00	10.07
NII	3367	231.53	987.84	0.08	33,121.21
TA	3330	40,795.96	123,570.50	0.64	1,700,000.00
TAGR	2933	20.33	177.41	0	8983.70
LOANRATIO	1499	60.67	16.44	0.05	185.58
LLPRATIO	3041	0.84	2.86	0.00	89.25
DEPOSITRATIO	3237	78.27	16.25	0.01	189.10
EQUITYRATIO	3330	8.46	9.96	0.03	131.44
NPL_TL	2010	5.89	7.21	0	93.60
GROWTH	288	6.06	4.01	− 13.13	26.63
INFLATION	288	0.00	4.24	− 24.06	96.55
MONEY	273	81.30	57.78	9.91	290.58
GOVT_EXP	260	97.67	12.82	48.31	119.43
2008–2020					
ZSCORE	9823	3.09	2.90	0.00	53.71
NII	9750	162.11	752.45	0.01	36,318.18
TA	9914	76,770.80	299,855.20	1.17	5,100,000.00
TAGR	9171	19.67	230.36	0.01	17,625.40
LOANRATIO	8192	58.64	36.14	0.07	2550.80
LLPRATIO	9419	0.57	1.54	0.00	117.41
DEPOSITRATIO	9467	77.23	13.62	0.20	152.02
EQUITYRATIO	9870	10.29	10.62	0.02	159.13
NPL_TL	7747	5.80	201.05	0	17,490.3
GROWTH	288	4.62	3.60	− 56.31	25.12
INFLATION	288	0.00	2.07	− 8.47	20.17
MONEY	281	110.20	75.89	24.29	452.54
GOVT_EXP	285	97.82	15.51	40.11	126.82

ZSCORE and TA are not logged in this table

It has been pointed out that the dependence of banks on noninterest incomes has come down after the 2008. Haubrich and Young (2019) shows this for the US banks wherein they indicate that noninterest income as a share of banks’ revenue has shown a declining trend. We see this for NII in Table 3. The average of NII over 1996–2007 was 231.53% which decreased to 162.11% during 2008–2020. The average of the ZSCORE, which is our dependent variable, has increased during 2008–2020 as compared to the first time period.

Even though we do not report the descriptive statistics for the COVID-afflicted year 2020, some of the statistics are worth noting. ZSCORE has decreased marginally to 2.99 while NII has risen sharply to 203.07 as normal avenues to earn revenues dried up (as reported by Li et al., 2021). Most bank-specific variables have not changed much but NPL_TL has risen to 7.93. GROWTH has collapsed to − 1.04 as would be expected while MONEY has risen to 169.98 and GOVT_EXP has risen to 99.40 as part of the efforts to support the economies of the affected countries.

Table 4 reports our estimated equations for ZSCORE for the two periods, 1996–2007 and 2008–2020. The results of Table 4 show that all the reported equations satisfy the requirements of a good GMM model (for details see Appendix 1).

**Table 4:** Bank risk-all countries

Row no.	Equation numbers	1996–2007	2008–2020
		(1)	(2)
1	ZSCORE(t − 1)	0.0159 (0.057)	0.2542*** (0.060)
2	NII	− 0.0002 (0.000)	− 0.0001*** (0.000)
3	TA	− 0.4115 (0.258)	− 0.4856*** (0.143)
4	TAGR	0.0002 (0.001)	− 0.0008 (0.001)
5	LOANRATIO	0.0521 (0.034)	− 0.0021 (0.010)
6	LLPRATIO	0.2603 (0.182)	0.0618** (0.030)
7	DEPOSITRATIO	− 0.0078 (0.016)	− 0.0187** (0.009)
8	EQUITYRATIO	− 0.0207 (0.043)	− 0.0652** (0.027)
9	NPL_TL	− 0.0689** (0.027)	− 0.0245 (0.023)
10	GROWTH	0.2187*** (0.065)	0.0497** (0.018)
11	INFLATION	− 0.0756* (0.045)	− 0.0288* (0.015)
12	MONEY	0.0216*** (0.007)	0.0076*** (0.002)
13	GOVT_EXP	0.0876 (0.067)	0.0565 (0.039)
14	CONSTANT	− 9.9272 (9.285)	0.4099 (3.048)
15	No. of Obs	1028	5538
16	No. of groups	280	745
17	Maximum number of time periods	10	13
18	Average number of time periods	3.67	7.43
19	No. of instruments	20	25
20	F	7.03*** [0.000]	69.34*** [0.000]
21	Hansen	8.93 [0.178]	9.04 [0.618]
22	Diff in Hansen	4.78 [0.311]	6.14 [0.189]
23	AR(2)	− 1.03 [0.304]	− 1.17 [0.242]

(1) *P < 0.10, **P < 0.05, ***P < 0.01 (2) numbers in parentheses are standard errors and the numbers in square brackets are *P*-values (3) The lagged dependent variable, TA, NII and NPL_TL are the instrumented variables while EQUITYRATIO, DEPOSITRATIO, GROWTH and Level of GDP in constant prices are the instrumental variables used (4) The average numebr of time periods is the number of observations divided by the number of groups

The coefficient of NII is seen to be negative and significant at 1% level in Eq. (2) but not in Eq. (1) for the pre-GFC period (N.B.: equation numbers are listed in the second row of Table 4 as well as in subsequent tables). Remembering that our dependent variable is logged and NII is not logged, Eq. (2) indicates that a 1 unit (in fact, one percentage point) decrease in NII will increase the ZSCORE by 0.01%. This is an important result which shows that lowered dependence on non-interest income improves the stability of banks. Among the other bank-related variables TA, LLPRATIO, DEPOSITRATIO and EQUITYRATIO are signficant in the post-GFC period. We have found that TA is negative and signficant which is in line with the results reported by Lee et al. (2014). Further, our reults for LLPRATIO, DEPOSITRATIO and EQUITYRATIO corroborate those of Lee et al. (2014). As far as EQUITYRATIO is concerned, the results of Lee et al. (2014) are not consistent with respect to sign while we have found the coefficient to be negative and significant in the second time period. Finally, the negative and signficant coefficient of NPL_TL is in line with that of Salike and Ao (2018).

Both GROWTH and INFLATION are seen to be statistically signficant in Eq. (2). GROWTH is seen to improve the ZSCORE in the both the time periods but INFLATION which has a negative coefficient worsens the risk. As far as GROWTH is concerned, our results agree with Salike and Ao (2018), which also is a multi-country study like ours but differ from their result regarding INFLATION which they found to be non-signficant while our results point to marginal signficance. On the other hand, Pham et al. (2021) found inflation to have a positive and signficant coefficient, albeit for a single country, namely, Vietnam. As far as the policy variables, MONEY and GOVT_EXP, are concerned we have found that MONEY is positive and significant in both the the time periods while GOVT_EXP does not seem to have a consequential impact on bank stability.

The fact that the coefficient of NII is consistently negative is in line with results obtained in the USA (see DeYoung & Rice, 2004; DeYoung & Roland, 2001; Stiroh, 2004, 2006; Stiroh & Rumble, 2006) and for Asian banks by Lee et al. (2014). However, our results differ from those of Baek et al. (2018) and Bian et al. (2015). It must be pointed out that none of these studies have examined the impact of NII on profitability on either side of the GFC of 2008. Interestingly, the results for German banks reported by Kohler (2014) show that banks that focus on lending and deposit-taking services become more stable if they increase the share of their non-interest income. Engle et al. (2014) also found that NII has a positive impact on ROE and ROA (depending on the level of concentration in the banking industry) but the z-score is negatively related to non-interest income in low concentration banking. Finally, our results corroborate those of Williams (2016) for Australia who found that non-interest income was riskier than interest income.

### Asia/Pacific Developed Countries

We now turn our attention to the developed countries of the Asia/Pacific region. We report in Table 5 the descriptive statistics for this group of countries but report these only for ZSCORE and NII (other descriptive statistics are available with the authors).

**Table 5:** Descriptive statistics of Asia/Pacific developed countries

	Frequencies	Mean	S.D	Min	Max
1996–2007					
ZSCORE	1.729	2.35	1.79	0.01	9.67
NII	1.696	292.40	1285.20	0.78	33,121.21
2008–2020					
ZSCORE	3.281	2.67	2.17	–	15.44
NII	3.066	187.40	512.00	0.19	16,218.75

Please see note to Table 3

The statistics of Table 5 show that as the average of NII has fallen from the first time period to the second one by 36%, the average ZSCORE has increased by 14%, suggesting a negative relationship between the two. However, within the group, there is variability: NII has fallen in Australia, Japan, South Korea and New Zealand but risen in Hong Kong and Singapore while ZSCORE has fallen for Australia, Hong Kong and Singapore but risen in Japan, South Korea and New Zealand. In Table 6, we will investigate whether this negative relationship between ZZSCORE and NII continues to hold. It may be mentioned (as discussed in connection with Table 2 in Sect. 2) that the Asia/Pacific Developed Countries group is defined in two ways: one, excluding Australia and New Zealand and, two, including these two countries. Table 6 reports results of this group excluding Australia and New Zealand but Appendix Table 12 reports results when we include Australia and New Zealand.

**Table 6:** Bank risk: Asia/Pacific developed countries (excluding Australia and New Zealand)

Equation numbers	1996–2007	2008–2020
	(3)	(4)
ZSCORE(t − 1)	0.3024 (0.196)	− 0.0338 (0.050)
NII	0.000002 (0.000)	− 0.0004*** (0.000)
TA	0.7617** (0.267)	− 0.2600* (0.136)
TAGR	0.0013 (0.002)	0.0137 (0.011)
LOANRATIO	− 0.0021 (0.026)	− 0.0619*** (0.018)
LLPRATIO	0.7336 (0.485)	0.7311 (0.613)
DEPOSITRATIO	0.1345** (0.055)	0.0129 (0.015)
EQUITYRATIO	0.1076** (0.040)	0.1183* (0.061)
NPL_TL	− 0.2039 (0.123)	0.0223 (0.107)
GROWTH	0.1650* (0.086)	0.0192 (0.016)
INFLATION	− 0.0796 (0.162)	− 0.0024 (0.024)
MONEY	− 0.0096 (0.007)	− 0.0047** (0.002)
GOVT_EXP	− 0.0369 (0.060)	0.0452 (0.030)
CONSTANT	− 13.5869* (7.661)	2.0387 (2.942)
No. of Obs	341	1476
No. of groups	98	160
Maximum number of time periods	10	13
Average number of time periods	3.48	9.22
No. of instruments	25	25
F	19.40*** [0.000]	18.19*** [0.000]
Hansen	13.99 [0.233]	9.94 [0.535]
Diff in Hansen	5.17 [0.270]	4.52 [0.340]
AR(2)	− 0.42 [0.677]	0.44 [0.659]

Please see notes to Table 4

The estimation results for the Asia/Pacific Developed counties show that the coefficient of NII is negative and significant in the second time period, that is, in the post-GFC time period. A one unit decrease in NII increases ZSCORE by 0.04%, values that are larger than those reported in Table 4. Hence, the fact that the average NII has decreased in the second time period points to an improvement in the stability of banks in the post-GFC time period. This result more or less exactly matches that obtained when we include Australia and New Zealand in this group (see Table 12 in the Appendix 2).

Our results (especially those in Appendix Table 12) seem to vindicate the Williams (2016) study on Australian Banks which showed that non-interest income is riskier than interest income. For Japan, Hong and Kandrac (2018) point to the peculiarity of negative interest rates leading to lower z-scores which forced banks to take more risks. The Reserve Bank of New Zealand (2019) points out that low interest rates induced banks to shift to non-interest revenues. Baek et al. (2018) have found for South Korea a negative impact of some components of non-interest incomes on z-scores.

As far as the coeffients of the other variables are concerned, TA has a negative impact on ZSCORES in Eqs. (3) and (4) though this result is replicated only in the post-GFC period in Table 12 when we include Australia and New Zealand. Among the other bank-related variables, DEPOSITRATIO and EQUITYRATIO are signficant in the pre-GFC period while LOANRATIO and EQUITYRATIO are signficant in the post-GFC period. Inclusion of Australia and New Zealand in the estimation changes the picture quite dramatically. None of the bank-related variables (other than NII and TA) are signficant in the post-GFC period (see Table 12 in the Appendix 2). However, in the pre-GFC period, EQUITYRATIO and NPL_TL are significant.

Considering the macroeconomic variables, GROWTH is marginally signficant in Eq. (3) while MONEY is negative and significant in Eq. (4). The fact that INFLATION is not signficant should not be surprising given that the literature states that bank stability may be affected only when inflation rises above a threshold level (Dhal et al., 2011). By and large, the average rate of inflation in this group of countries has been around 1.5% or lower which may not impact bank stability in a signficant manner.

### East and South-east Asian Countries

This section focuses attention on the countries in East and South-east Asia. Table 7 reports the basic descriptive statistics for this group of countries.

**Table 7:** Descriptive statistics of east and south-east Asian countries

	Frequencies	Mean	S.D	Min	Max
2000–2007					
ZSCORE	1228	2.11	1.89	0.002	10.07
NII	1216	154.49	378.70	0.085	10,325.00
2008–2018					
ZSCORE	5491	3.46	3.36	0.00	53.71
NII	5427	138.54	859.14	0.005	36,318.18

Please see note to Table 3

The statistics of Table 7 show that as the average of NII has fallen from the first time period to the second one by 10%, the average ZSCORE has increased by 63%, suggesting a negative relationship between the two. In this group of 12 countries, NII has risen for five countries (Brunei, China, Indonesia, Laos and Thailand) and fallen in the other 6 countries (Cambodia, Malaysia, Mongolia, Philippines, Taiwan and Vietnam) There was no data for Macau for the first time-period and hence change in its NII has not been computed. On the other hand, ZSCORE has risen in Brunei, China, Indonesia, Laos, Malaysia, Philippines, Taiwan, Thailand and Vietnam and fallen in Cambodia and Mongolia. In Table 8, we will investigate this relationship between NII and ZSCORE further.

**Table 8:** Bank risk: east and south-east Asian countries

Equation numbers	1996–2007	2008–2020
	(5)	(6)
ZSCORE(t − 1)	0.2717 (0.173)	0.3605*** (0.082)
NII	− 0.0007 (0.001)	− 0.0001*** (0.000)
TA	− 0.2700 (0.270)	0.2643* (0.147)
TAGR	− 0.0001 (0.001)	0.0022 (0.002)
LOANRATIO	− 0.0436** (0.020)	− 0.0318** (0.015)
LLPRATIO	0.1348 (0.118)	0.0918* (0.051)
DEPOSITRATIO	− 0.0219 (0.015)	0.0255** (0.011)
EQUITYRATIO	− 0.0227 (0.037)	0.0657** (0.033)
NPL_TL	− 0.0031 (0.0122)	− 0.0007 (0.003)
GROWTH	0.0948 (0.086)	− 0.0121 (0.024)
INFLATION	− 0.0064 (0.021)	0.0209** (0.010)
MONEY	0.0134** (0.007)	− 0.0005 (0.002)
GOVT_EXP	− 0.0330 (0.48)	− 0.0590** (0.027)
CONSTANT	8.4403 (7.651)	3.0771 (1.992)
No. of Obs	404	2732
No. of groups	106	456
Maximum number of time periods	8	13
Average number of time periods	3.81	5.99
No. of instruments	29	20
F	7.83*** [0.000]	71.16*** [0.000]
Hansen	13.48 [0.565]	9.02 [0.172]
Diff in Hansen	6.72 [0.567]	7.50 [0.112]
AR(2)	1.15 [0.252]	0.82 [0.412]

Please see notes to Table 4

The coefficients of NII continue to be negative and significant in the post-GFC period but they are smaller than those seen in Table 6 for Asia/Pacific Developed Countries. Hence, even for countries in East and South-east Asia, a reduction in the dependence on non-interest income makes the banks less risky. Given that the share of non-interest income has fallen in the second time period, we can conclude that banks have become more stable in the post-GFC period. Our results differ from those obtained by Hsieh et al. (2013) who find the direct effects of NII on z-score to be positive but it is important to note that the time period for that study ended with 2009. The results of Lee et al. (2014) differ according to whether profitability or risk is being estimated. NII tends to increase profitability and decrease risk for commercial banks but not for savings banks. Further, NII increases risk in high income countries, but benefits banks in middle and low income countries through improving profitability or reducing risk. TA has a marginally significant and negative relationship with ZSCORE in Eq. (6) for the post-GFC time period. Among the other bank-related variables, we find LOANRATIO is the only signficant variable in the pre-GFC period while LOANRATIO, LLPRATIO, DEPOSITRATIO and EQUITYRATIO are signficant in the post-GFC period. As far as the macroeconomics variables are concerned, INFLATION is positive and signficant in Eq. (6) for the post-GFC period. Unlike the results of Table 6, the coefficient of INFLATION is positive indicating an improvement in bank stability. Of course, it must be remembered that average INFLATION in this group of countries has been moderate: 3.51% in the pre-GFC time period and lower at 2.83% in the post-GFC. Among the policy variables, MONEY is positive and signficant in the pre-GFC period while GOVT_EXP is negative and signficant in the post-GFC period.

### South Asian Countries

We now consider the last group in our dataset. Table 9 provides the descriptive statistics for this group of countries.

**Table 9:** Descriptive Statistics for South Asian Countries

	Frequencies	Mean	S.D	Min	Max
1996–2007					
ZSCORE	369	2.19	1.71	0.01	7.44
NII	455	210.58	808.82	0.29	11,753.85
2008–2020					
ZSCORE	1051	2.44	1.77	0.04	15.87
NII	1257	202.21	748.75	1.80	18,060.00

Please see note to Table 3

The ZSCORE for this group of countries is lower than that seen for the previous two sub-groups, especially in the post GFC time period. There is only a marginal decline in the value of NII while it has declined by 36% for countries in the Asia/Pacific region and by 10% for countries in the East and South-east of Asia. In this group of 6 countries, NII has risen in India while it has declined in Bangladesh, Nepal, Pakistan and Sri Lanka (data for Maldives was missing for the pre-GFC time period). On the other hand, ZSCORE has fallen in India and Pakistan and risen in Bangladesh, Nepal and Sri Lanka. Table 10 shows the estimated equation for South Asian countries.

**Table 10:** Bank risk: south Asian countries

	1996–2007	2008–2020
Equation numbers	(7)	(8)
ZSCORE(t − 1)	0.1476 (0.109)	0.5468*** (0.051)
NII	− 0.0044*** (0.001)	− 0.0002** (0.000)
TA	0.4473** (0.191)	0.2137*** (0.056)
TAGR	0.0120*** (0.003)	0.0103** (0.004)
LOANRATIO	0.0531** (0.022)	0.0103* (0.006)
LLPRATIO	0.1763 (0.162)	0.1150 (0.079)
DEPOSITRATIO	0.0001 (0.013)	0.0016 (0.005)
EQUITYRATIO	− 0.0400 (0.041)	0.0139** (0.007)
NPL_TL	0.0471** (0.018)	− 0.0142** (0.007)
GROWTH	− 0.0882 (0.087)	0.0088 (0.0165)
INFLATION	− 0.0233* (0.013)	− 0.0057 (0.009)
MONEY	0.0049 (0.021)	− 0.0227** (0.009)
GOVT_EXP	− 0.0096 (0.024)	− 0.0327* (0.018)
CONSTANT	− 4.3251 (2.778)	2.3419 (2.219)
No. of Obs	141	781
No. of groups	46	74
Maximum number of time periods	6	13
Average number of time periods	3.07	10.55
No. of instruments	29	34
F	114.97*** [0.000]	170.72*** [0.000]
Hansen	9.84 [0.830]	22.46 [0.316]
Diff in Hansen	8.26 [0.409]	5.97 [0.651]
AR(2)	− 0.71 [0.479]	− 0.02 [0.985]

Please see notes to Table 4

NII is seen to be negative and highly significant during both the time periods in Eqs. (7) and (8). The coefficient of NII is much smaller in the post-GFC period indicating its decreased imprortance. Our results, though not directly comparable, differ from those of Ahamed (2017) who finds returns as well as risk-adjusted returns to be positively related to NII. Compared to the other sub-groups, a much larger number of bank-related variables are signficant for South Asian countries. Thus, TA, TAGR, LOANRATIO and NPL_TL are signficant in both the time periods while EQUITYRATIO is significant only in the post-GFC period. It is notable that the sign of the coefficient of TA for this sub-group is different from the previous two sub-groups. For South Asian countries, an increase in TA improves ZSCORE while it lowered it in the previous sub-groups. Among the macroeconomic variables, INFLATION is both negative and marginally significant in the pre-GFC time period. Finally, both the policy variables are seen to be significant in the post-GFC period.

## Further Discussion of Results

**Fig. 1:**
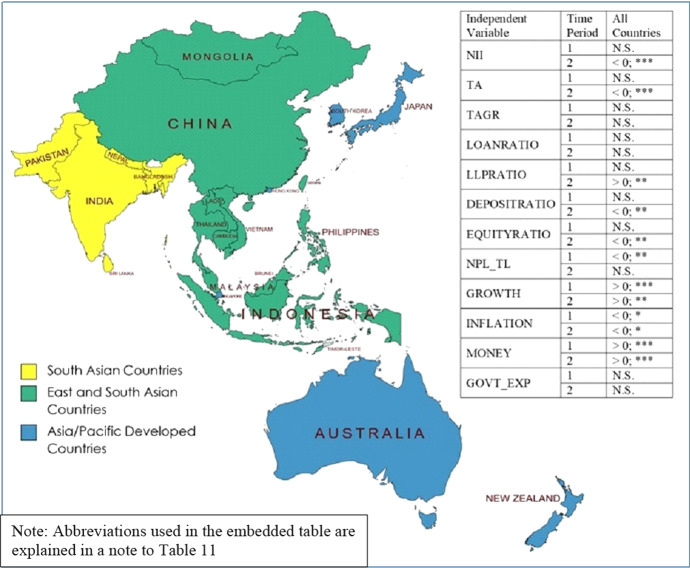
All Countries

Having estimated equations for all countries in our dataset as well as for sub-groups of these countries, we now bring together all our results for the purpose of comparison. We present in Fig. 1 a map of all the countries included in our data set (created using the tool at mapchart.net) which also colour codes the sub-groups of countries that we have created. Embedded in Fig. 1 are the summarised results of Eqs. (1) and (2) from Table 4 for all countries.

In Table 11, we report on the results of the sub-groups of countries. In this table, we include the results of Eqs. (3) and (4) from Table 6, Eqs. (5) and (6) from Table 8, Eqs. (7) and (8) from Table 10 and equations (A1) and (A2) from Appendix Table 12. Since detailed results are available in earlier tables, in Table 11 we report only the significance or otherwise of the coefficients and, if signficant, their signs are also reported.

**Table 11:** Comparison of results across sub-groups of countries

Independent variable	Time period	Asia/Pacific developed (A)	Asia/Pacific developed (B)	East and S.E. Asia	South Asia
NII	1	N.S	N.S	N.S	< 0;***
	2	< 0;***	< 0;***	< 0;***	< 0;**
TA	1	> 0;**	N.S	N.S	> 0;**
	2	< 0;*	< 0;**	> 0;*	> 0;**
TAGR	1	N.S	N.S	N.S	> 0;***
	2	N.S	N.S	N.S	> 0;**
LOANRATIO	1	N.S	N.S	< 0;**	> 0;**
	2	< 0;***	N.S	< 0;**	> 0;**
LLPRATIO	1	N.S	N.S	N.S	N.S
	2	N.S	N.S	> 0;*	N.S
DEPOSITRATIO	1	> 0;**	N.S	N.S	N.S
	2	N.S	N.S	> 0;**	N.S
EQUITYRATIO	1	> 0;**	> 0;*	N.S	N.S
	2	> 0;*	N.S	> 0;**	> 0;**
NPL_TL	1	N.S	< 0;***	N.S	> 0;**
	2	N.S	N.S	N.S	< 0;**
GROWTH	1	> 0;*	N.S	N.S	N.S
	2	N.S	N.S	N.S	N.S
INFLATION	1	N.S	N.S	N.S	< 0;*
	2	N.S	N.S	> 0;**	N.S
MONEY	1	N.S	N.S	> 0;**	N.S
	2	< 0;**	N.S	N.S	< 0;**
GOVT_EXP	1	N.S	N.S	N.S	N.S
	2	N.S	N.S	< 0;**	< 0;*

Time Period 1 refers to pre-GFC period (1996–2007) and Time Period 2 refers to post-GFC period (2008–2020)

Asia/Pacific Developed (A) excludes Australia and New Zealand and Asia/Pacific Developed (B) includes Australia and New Zealand

The differences in results reported in Table 11 justifies our approach of estimating equations for various sub-groups of countries. This diversity would have been lost had we confined ourselves to the overall group of 24 countries. One result that carries across all sub-groups is the one related to NII. Our results clearly show that in the post-GFC period, diversifying income (as captured by NII) reduces the stability of the banks. For South Asia, this results holds in the both the time periods. Size of banks as reflected by the variable TA has an ambiguous relationship with bank stability. IMF (2014) distinguishes between individual bank risk and systemic risk. The perception that large banks are “too big to fail” and will be bailed out leads to a moral hazard problem leading the banks to take more risks. Our results seem to bear this out for the group of All Countries and Asia/Pacific Developed Countries (A and B) in the post-GFC period. This is not surprising since in the Asia/Pacific Developed Countries, top four banks account for a very large market share in terms of Assets: in almost all countries, the top four banks account for more than 50% market share with Australia the highest at 77% (Dahl et al., 2019). IMF (2014) also states that large banks are able to diversify risks better which reduces risks and hence TA will be positively associated with ZSCORE.  This appears to be true for South Asian banks in both the time periods and Asia/Pacific Developed (A) in the pre-GFC period. Further, rate of growth of TA (TAGR) plays a role only in the South Asia group.

Among the other bank-specific variables, LOANRATIO plays a negative role in Asia/Pacific Developed (A) Countries and East and South-east Asian Countries but a positive role in South Asian countries. Kohler (2012) has reported that higher credit growth (which will be reflected in the LOANRATIO) increases bank risk. However, in the largest economy in South Asia, namely, India, the there is a history of bailout of banks by the government when loans turn bad (Parkin & Kazmin, 2020). This safety net results in a contrary relationship between LOANRATIO and bank risk in South Asian countries. LLPRATIO is non-significant in most country sub-groups while DEPOSITRATIO is negative in the post-GFC time period when all countries are considered but positive for East and South-east Asian countries. EQUITYRATIO is positive for many sub-groups of countries in the post-GFC period. Finally, NPL_TL has a negative impact on bank stability except for South Asian countries in the pre-GFC period.

Both the macroeconomic variables, GROWTH and INFLATION, are signficant for the All Countries group with GROWTH improving bank stability and INFLATION lowering it. It is important to note that when all countries are considered together, both the macroeconomic variables show consistent results before and after the GFC but the effect of these two variables is not seen when we consider sub-groups of countries. A possible explanation for this is that, when all countries are considered, there is sufficient variablity among the countries as far as GROWTH and INFLATION is concerned which is not the case when sub-groups of countries are considered. For instance, the standard deviation of GROWTH when all countries are taken together is higher than for each of the sub-groups and likewise for INFLATION barring East and South-east Asian countries which show higher variability. Among the policy variables, MONEY is seen to be positive at the All Countries level in both the time periods but is negative for Asia/Pacific Developed (A) countries and South Asian countries in the post-GFC period. GOVT_EXP is marginally significant in the countries of East and South-east Asia and South Asia.

### Comparison with non-Asian Countries

In this sub-section, we seek to relate our results for Asian banks to results obtained in other regions of the world. Since the primary focus of this paper has been the effect of income diversification on bank stability, we first look at the studies in other countries with respect to the inpact of NII. We find that, uniformly, for most studies in the USA, NII has a negative impact on bank stability (See Stiroh, 2004; Stiroh & Rumble, 2006; Brunnermeier et al., 2020 and Kim & Kim, 2020). One study covering OECD countries has found a negative impact of NII (Kim et al., 2020) while another found no effect (Hahm, 2008). However, Smith et al.. (2003), Baele et al. (2007), Lepitit et al. (2008) and Kohler (2014) have found a positive impact of NII. Considering banks from Africa, Alhassan (2015) found for Ghana that income diversification does not support profits. Finally, Sharma and Anand (2018) find for BRICS countries a positive relationship between income diversification and bank risk though they caution that indiscriminate diversification may not be efficient and may lead to increased risk and reduced returns.

As far as the other bank-specific variables were concerned, only TA was present in most of the estimated models reported by other studies. The evidence for TA is mixed: two studies for the USA found a positive effect (Stiroh, 2004 and Stiroh & Rumble, 2006), while Brunnmeier et al. (2020) and Kohler (2014) found a negative effect. Only three studies had considered TAGR and the effect was seen to be negative in two of those (Stiroh, 2004 and Stiroh & Rumble, 2006). Among the other bank-specific variables EQUITYRATIO has been employed by a few studies, two of which found a positive effect (Stiroh, 2004 and Stiroh & Rumble, 2006) while two others found it to be non-significant. Only Hahm (2008) had included some macroeconomic variables GROWTH and INFLATION in the estimated equation, both of which were found to be non-signficant.

### Policy Implications

From the point of income diversification, the most signficant policy implication that emerges is that banks need to proceed with caution. The fact that the sign and signficance of NII is maintained in all our estimated equations points to the robustness of our result. While there have been occasional instances of such diversification being beneficial for risk reduction, by and large, our results suggest this is not the case. We have reported consistent results that income diversification and bank risk/stability are negatively related. This result is very important given the current prevalence of the COVID-19 pandemic. As stated in connection with Table 3 in Sect. 3.1, the value of NII has increased in the year 2020. Banks, in order to protect their revenues, might increase their dependence on non-interest income which would impact negatively on bank stability. Since this is still an evolving situation, the exact contuours of the emerging situation are still fuzzy and it is not possible make definitive statements. Despite this, Li et al. (2021) report that while a shift to non-interest income might improve performance, it will increase risk.

From a macroeconomics policy point of view, monetary policy (as captured by MONEY) plays a far more important role as far as bank stability is concerned. An increase in money supply seems to have a positive impact on bank stability when we consider all countries together. As stated earlier, the traditional transmission channel of monetary policy, which was the norm pre-GFC, depended on the central banks control over reserve requirements and the money multiplier (Albertazzi et al., 2021). Post-GFC, however, unconventional monetary policy approaches emerged among which was helping banks avoid liquidity and funding difficulties  (Albertazzi et al., 2021). Central Banks in Asia have been providing such support to banks in the post-GFC period as well as in the aftermath of the COVID-19 pandemic (Barua & Samaddar, 2020). Our results show that such monetary policy measures have had a beneficial effect on bank stability. However, government expenditures do not seem to affect banks in the same way. While fiscal policy may have a role to play in stabilising an economy and in boosting growth, its direct impact on the banking system seems to be limited.

## Conclusion

The role of noninterest income in determining bank risk has been an active area of research especially for the USA and for Europe. By and large, studies have found that diversification away from traditional sources earnings has worsened bank risk (this has generally been true of studies related to the USA) while others have found the reverse (this has been true of studies related to European banks). This paper was concerned with examining bank risk for Asian banks. We have provided arguments that banking in Asia needs to be explored more deeply in view of significant differences between American/European banks and Asian banks.

Studies on Asian banks have not yet provided a coherent view on the impact of non-interest incomes on bank risk. While some studies have reported a worsening of bank risk due to income diversification towards non-interest incomes others have reported a lowering of risk. The latter studies have called for a further diversification of bank incomes as a way of improving bank stability. Our results cast doubt on this policy prescription. Further, our exercises have demonstrated the usefulness of adopting a granular approach to estimating bank stability. Confining our estimation to the aggregate level would have likely prevented us from examining differences across regions. We have also been able to demonstrate that we need to take account of the global GFC of 2008 in studying the impact of NII on bank risk given that this impact certainly changed as compared to the time period before the GFC.

To summarise the main result of our paper, we can say that there is reasonable unanimity across groups of countries that diversification of income hurts bank stability. It is interesting that, despite differences among these groups and among the countries within the groups, there is a clear message that an emphasis on NII does not render banks more stable. This result has become stronger in the post-GFC time period. It is also seen from the summary statistics that in all the groups the level of NII has come down in the post-GFC period which would suggest that banks would have gained in stability.

There are some important directions in which this research needs to be extended further. The role of macroeconomic variables needs to be explored further. We have stated that inflation above a certain threshold is likely to impact bank stability but the exact threshold for every group of countries or, indeed, every country needs to be determined. The role of policy variables, especially monetary policy, needs further explication especially since there is a great diversity among countries regarding the conduct of this policy. Finally, the most signficant direction in which research needs to extended is to explore the implications of the COVID-19 pandemic on bank stability. Since the full effects of the pandemic are still being played out in many countries, it might need some time for reliable data to be generated for such analysis to be carried out.

## Dynamic Panel Data Estimation

We wish to estimate a dynamic panel data model relating bank risk (ZSCORE) with NII in the presence of a set of control variables listed in Table 2.  We follow the approach of Arellano and Bond (1991) and the description given by (Roodman, 2009a, 2009b). below:

Our dynamic panel data model is given by:

A1.1 $$\begin{aligned} & ZSCORE_{it} = \alpha ZSCORE_{i,t - 1} + {\varvec{x}}_{it}^{{\prime }} {\varvec{\beta}} + e_{it} \\ & e_{it} = \, \mu_{i} + \, u_{it} \\ \end{aligned}$$

where *i* relates to the cross-section unit and *t* relates to time; ***x*** is a vector of financial variables and macroeconomic variables used as controls; e_it_, the disturbance is made up fixed effects, *µ*_*i*_ and shocks, u_*it*_. *α* and vector ***β*** are parameters.

*e*_*it*_ is taken to be a composite disturbance term made up of *µ*_*i*_ which are unobserved effect or unobserved heterogeneity related to each cross section unit and *u*_*it*_ is idiosyncratic error or time-varying error and represents unobserved factors that change over time and affect *ZSCOREit* and which are assumed to be independently and identically distributed (*i.i.d*). *µ*_*i*_ and *u*_*it*_ have means of zero and the correlation between the two is assumed to be zero given that *µ*_*i*_ are constant over time.

$$E\left( {\mu_{i} } \right) = E\left( {u_{it} } \right) = E\left( {\mu_{i} ,u_{it} } \right) = 0$$

The model given above has been estimated using two-step System-GMM. We guard against the warning of instrument proliferation by Roodman (2009a) by ensuring that, for each our equations, the time dimension is much smaller than the number of cross-section units. To evaluate the estimated equations, we report three tests: (a) Hansen’s test of over identifying restrictions (b) Difference-in-Hansen test of exogeneity of instrument subsets (c) Autocorrelation (AR2) Test. For the two Hansen tests, accepting the null hypotheses indicates that instruments are valid while accepting the null hypothesis for the third test indicates absence of autocorrelation.

## Appendix 2

See Table 12.

**Table 12:** Bank risk: Asia/Pacific developed countries (including Australia and New Zealand)

	1996–2007	2008–2020
Equation numbers	(A1)	(A2)
ZSCORE(t − 1)	0.0654 (0.179)	0.1999 (0.127)
NII	− 0.00003 (0.000)	− 0.0005*** (0.000)
TA	0.2906 (0.282)	− 0.6825** (0.301)
TAGR	0.0009 (0.002)	0.0015 (0.005)
LOANRATIO	− 0.0211 (0.019)	0.0482 (0.061)
LLPRATIO	0.4556 (0.491)	− 1.9508 (2.944)
DEPOSITRATIO	0.0708 (0.063)	− 0.0632 (0.045)
EQUITYRATIO	0.0774* (0.045)	0.0329 (0.067)
NPL_TL	− 0.2646*** (0.090)	0.4204 (0.398)
GROWTH	0.0954 (0.121)	− 0.0322 (0.050)
INFLATION	0.0283 (0.082)	− 0.0156 (0.038)
MONEY	− 0.0053 (0.010)	0.0019 (0.003)
GOVT_EXP	0.0985 (0.086)	− 0.0361 (0.067)
CONSTANT	− 14.8928** (7.179)	12.4275 (9.819)
No. of Obs	390	1744
No. of groups	108	194
Maximum number of time periods	10	13
Average number of time periods	3.61	8.99
No. of instruments	24	20
F	20.16*** [0.000]	18.86*** [0.000]
Hansen	11.24 [0.339]	6.95 [0.325]
Diff in Hansen	4.85 [0.183]	4.71 [0.318]
AR(2)	− 0.18 [0.860]	0.45 [0.654]

Please see notes to Table 4
